# Supplementary material for: Phase I Study of a Multivalent WT1 Peptide Vaccine (Galinpepimut-S) in Combination with Nivolumab in Patients with WT1-Expressing Ovarian Cancer in Second or Third Remission
Source: Cancers (Basel). 2023 Feb 25;15(5):1458. doi: 10.3390/cancers15051458 (PMC10001251; doi:10.3390/cancers15051458)
Supplement: Supplementary file 1 [file cancers-15-01458-s001.zip › cancers-2201866-supplementary/cancers-2201866-supplementary.pdf]

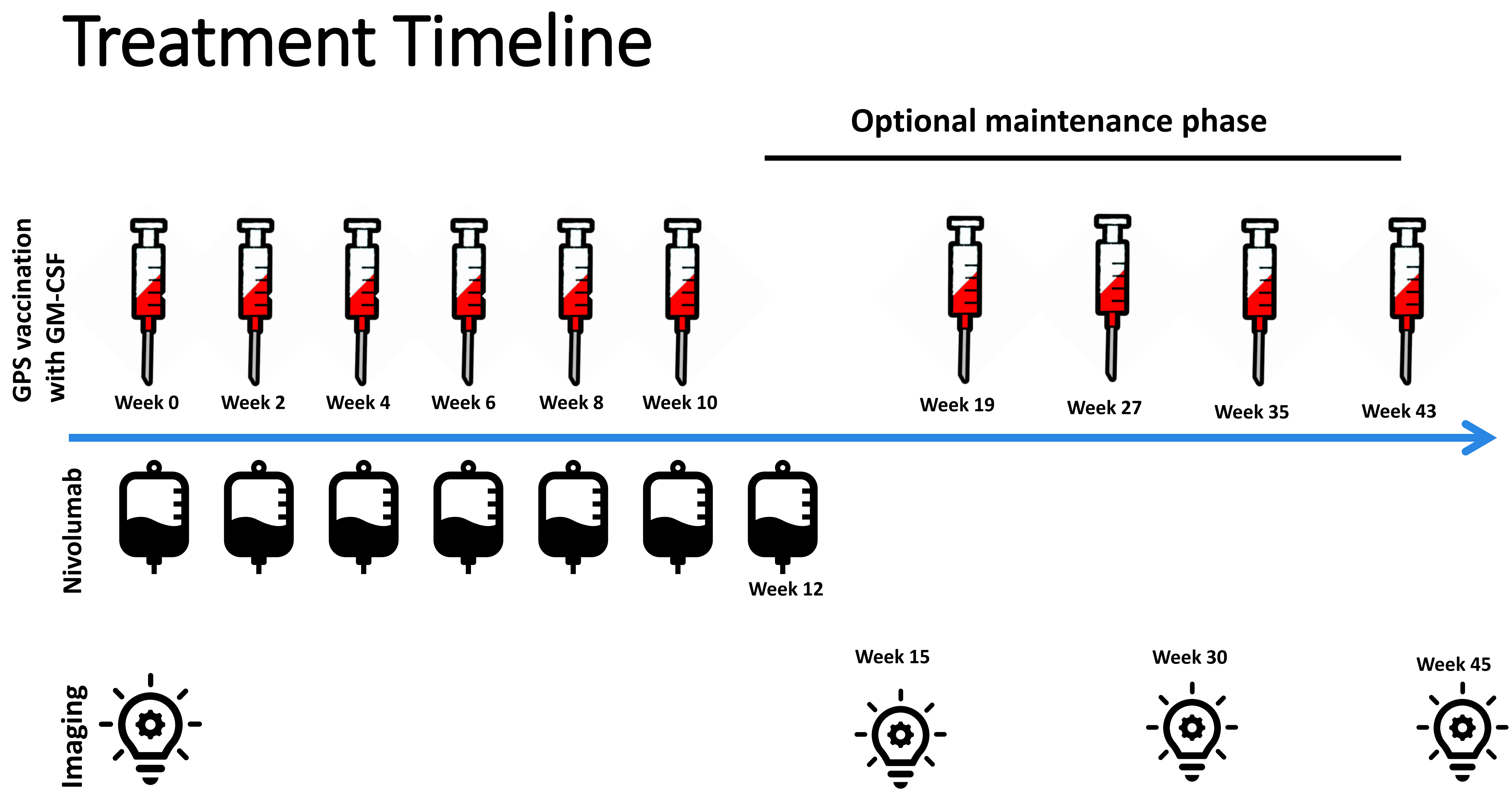

Figure S1: Treatment timeline. GPS, galinpepimut-S; GM-CSF, granulocyte-macrophage colony-stimulating factor

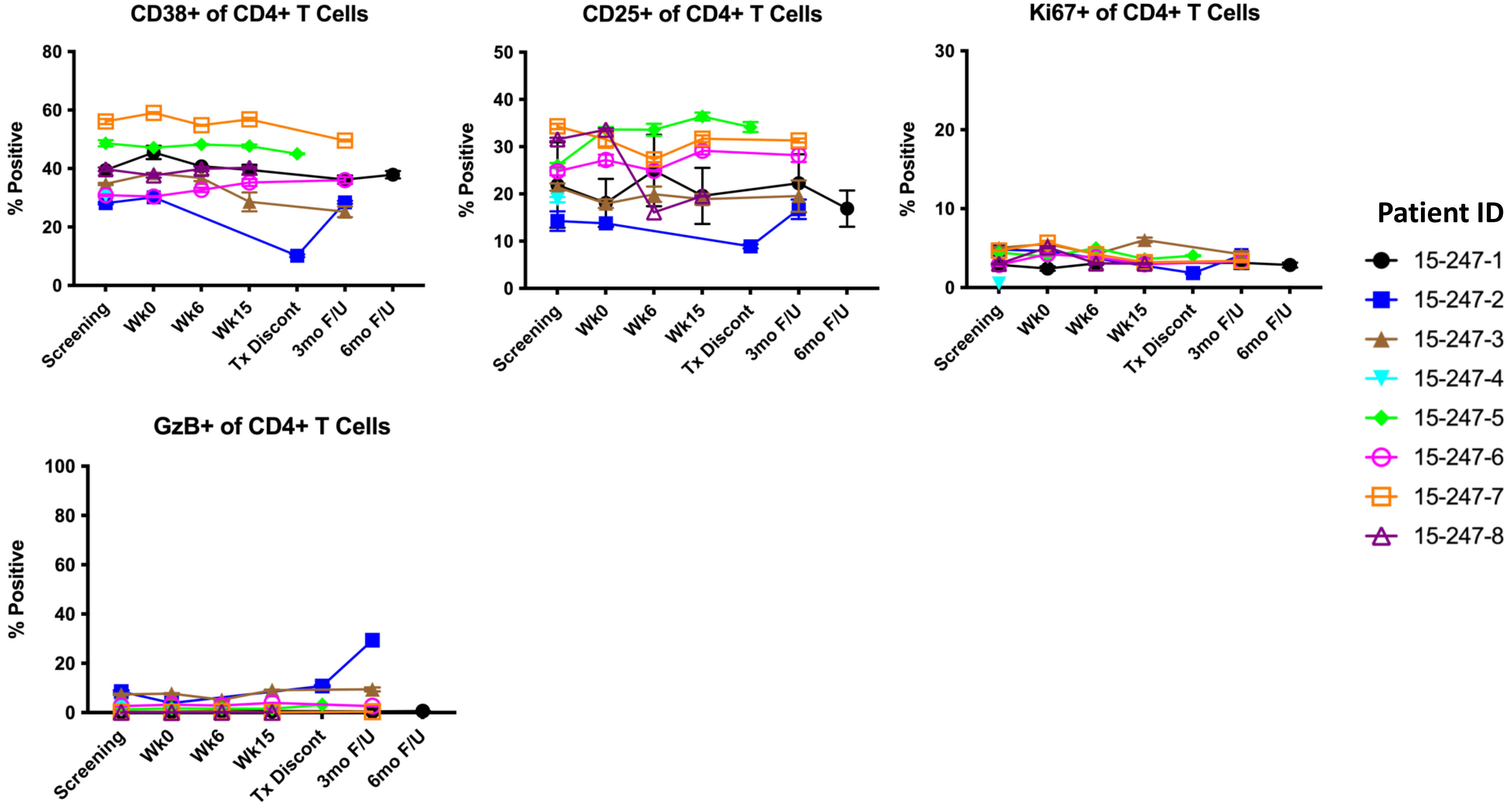

Figure S2: Summary of CD4+ cell subpopulation frequencies over time

GzB, Granzyme B

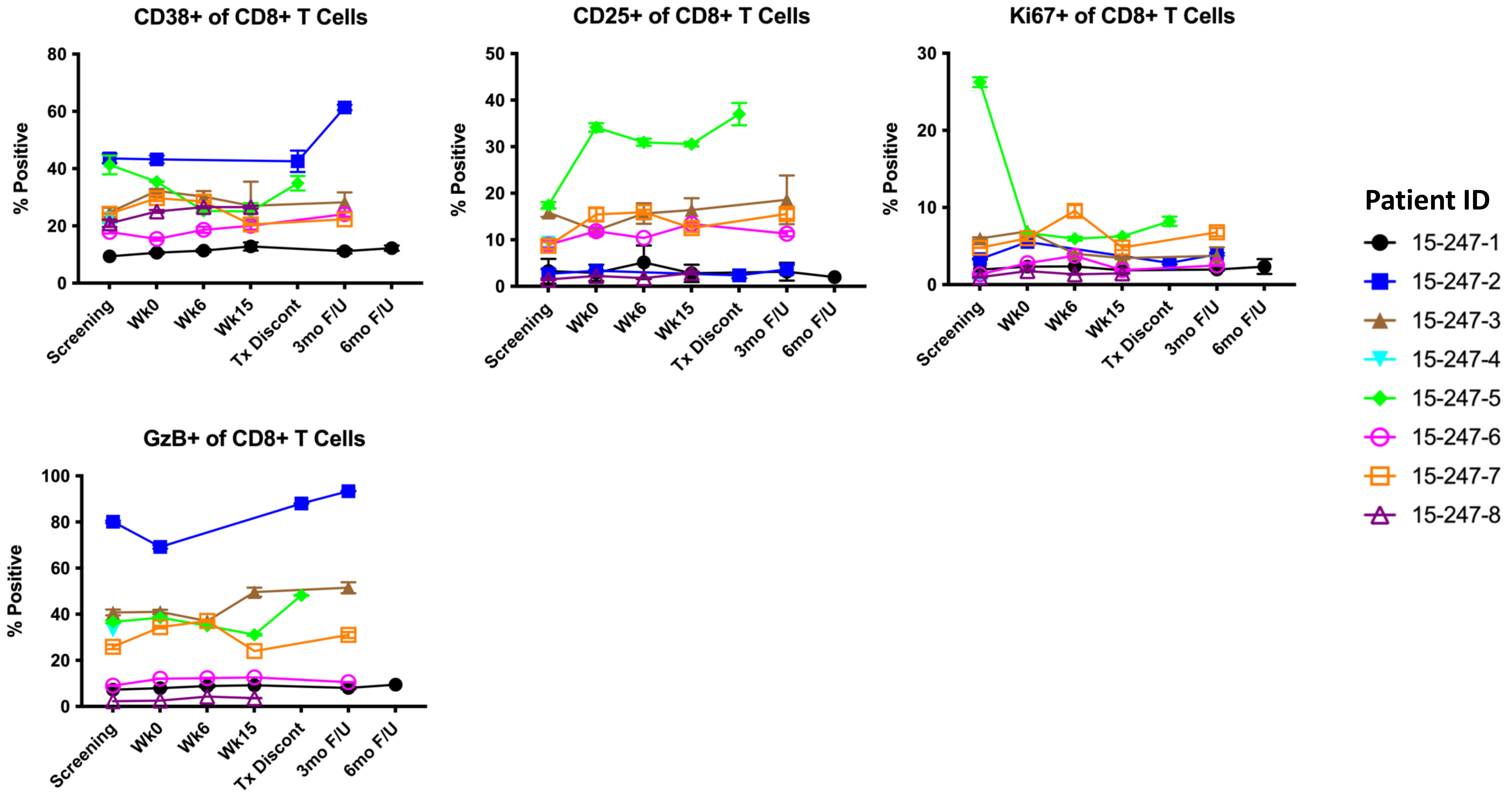

Figure S3: Summary of CD8+ cell subpopulation frequencies

GzB, Granzyme B

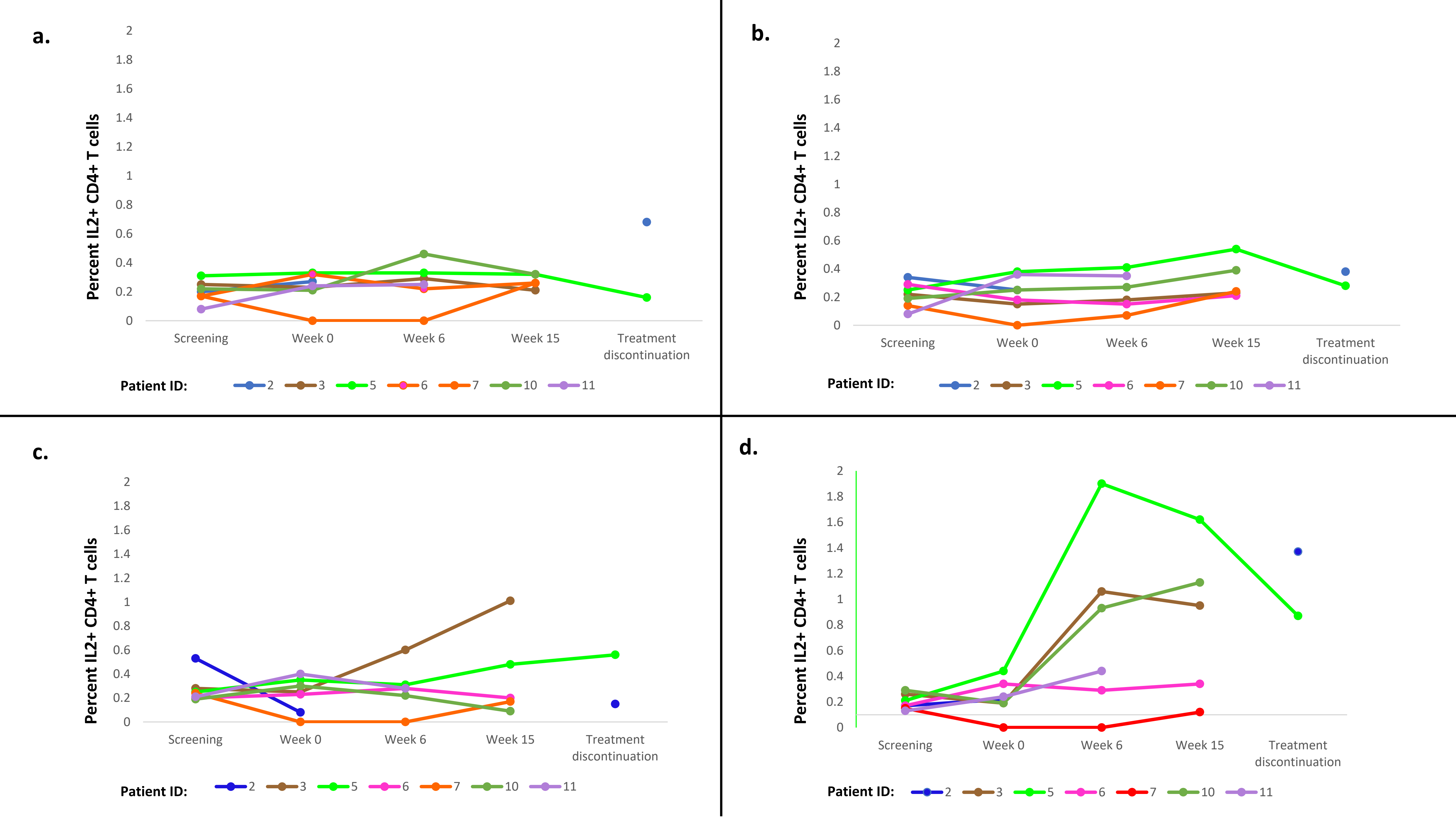

a.
Percent IL2+ CD4+ T cells
2
1.8
1.6
1.4
1.2
1
0.8
0.6
0.4
0.2
0
Screening
Week 0
Week 6
Week 15
Treatment discontinuation
Patient ID:
2
3
5
6
7
10
11
b.
Percent IL2+ CD4+ T cells
Screening
Week 0
Week 6
Week 15
Treatment discontinuation
Patient ID:
2
3
5
6
7
10
11
c.
Percent IL2+ CD4+ T cells
Screening
Week 0
Week 6
Week 15
Treatment discontinuation
Patient ID:
2
3
5
6
7
10
11
d.
Percent IL2+ CD4+ T cells
Screening
Week 0
Week 6
Week 15
Treatment discontinuation
Patient ID:
2
3
5
6
7
10
11

Figure S4: Cytokine responses of CD4 T cells to test peptides. (**a**) CD4 immune response against 122A in non-HLA2 patients (IL-2); (**b**) CD4 immune response against 122A1 in non-HLA2 patients (IL-2); (**c**) CD4 immune response against 427L in non-HLA2 patients (IL-2); (**d**) CD4 immune response against 331L in non-HLA2 patients (IL-2)

HLA, human leukocyte antigen; IL-2, interleukin 2

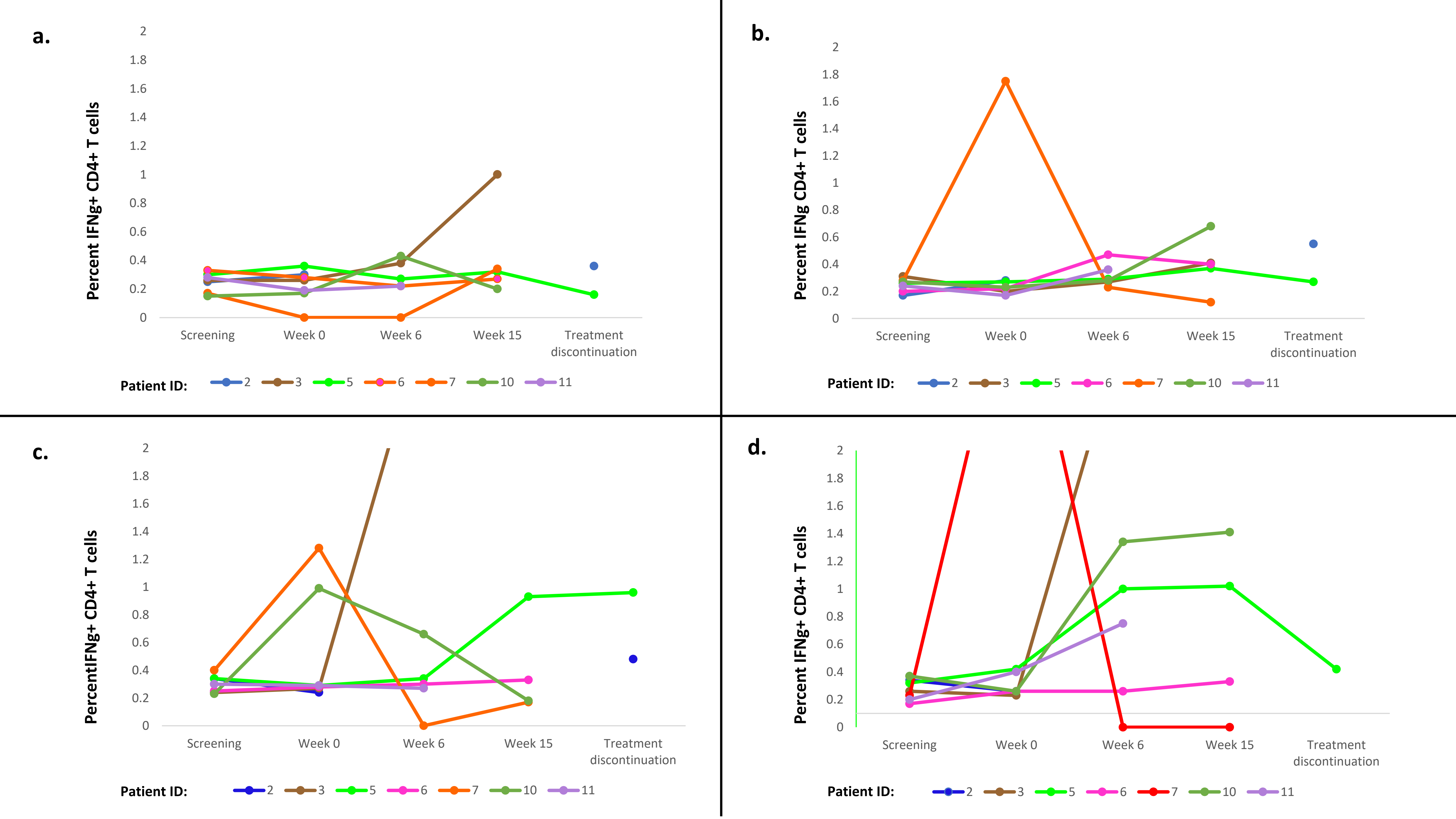

a.
Percent IFNg+ CD4+ T cells
2
1.8
1.6
1.4
1.2
1
0.8
0.6
0.4
0.2
0
Screening
Week 0
Week 6
Week 15
Treatment discontinuation
Patient ID:
2
3
5
6
7
10
11
b.
Percent IFNg CD4+ T cells
Screening
Week 0
Week 6
Week 15
Treatment discontinuation
Patient ID:
c.
PercentIFNg+ CD4+ T cells
Screening
Week 0
Week 6
Week 15
Treatment discontinuation
Patient ID:
d.
Percent IFNg+ CD4+ T cells
Screening
Week 0
Week 6
Week 15
Treatment discontinuation
Patient ID:

Figure S5: Cytokine responses of CD4 T cells to test peptides. (**a**) CD4 immune response against 122A in non-HLA2 patients (IFNɣ); (**b**) CD4 immune response against 122A1 in non-HLA2 patients (IFNɣ); (**c**) CD4 immune response against 427L in non-HLA2 patients (IFNɣ); (**d**) CD4 immune response against 331L in non-HLA2 patients (IFNɣ)

HLA, human leukocyte antigen; IFNɣ, interferon gamma

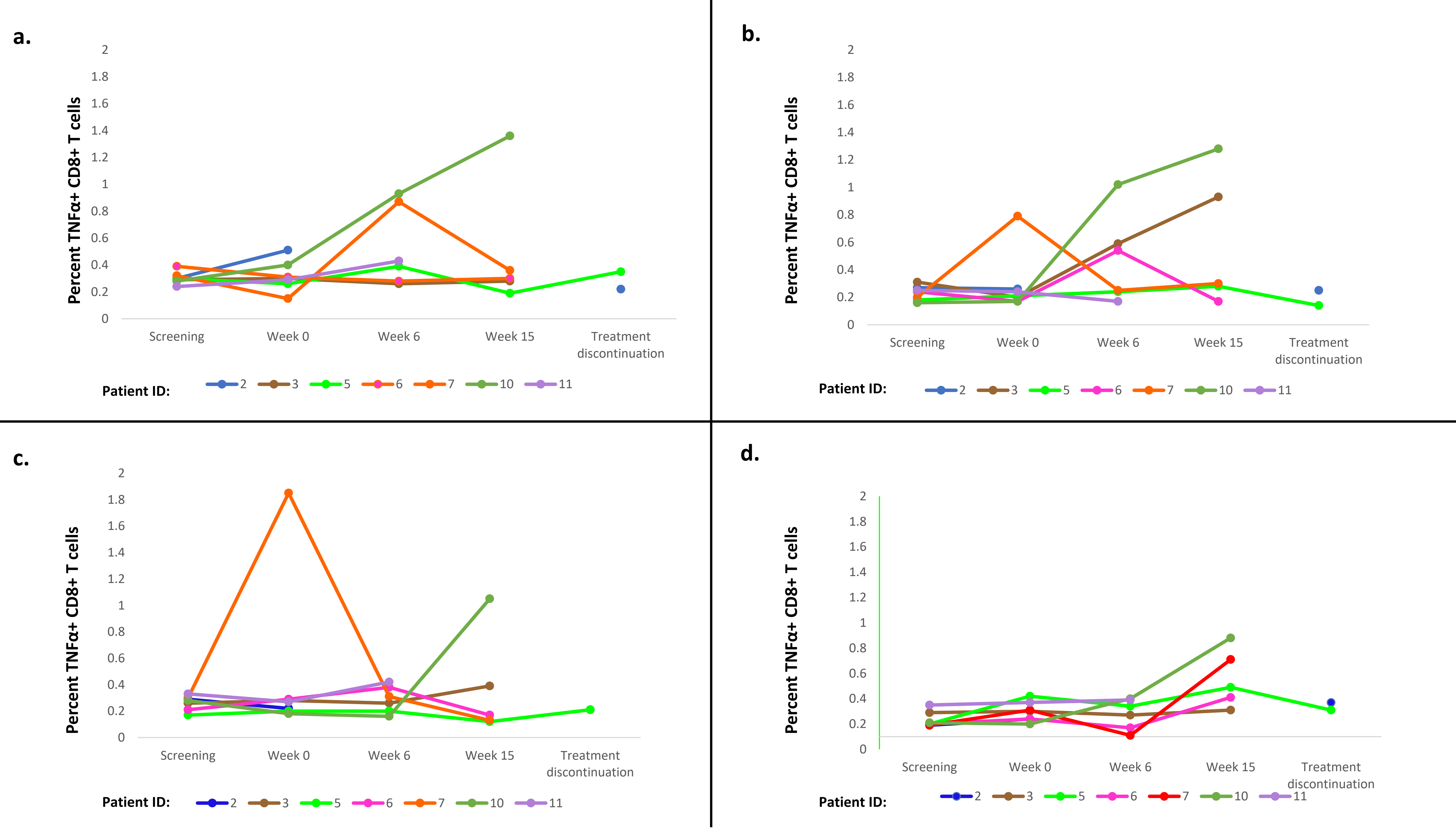
a.
b.
c.
d.
Percent TNFα+ CD8+ T cells
2
1.8
1.6
1.4
1.2
1
0.8
0.6
0.4
0.2
0
Screening
Week 0
Week 6
Week 15
Treatment discontinuation
Patient ID:
2
3
5
6
7
10
11

Figure S6: Cytokine responses of CD8 T cells to test peptides. (**a**) CD8 immune response against 122A in non-HLA2 patients (TNFα); (**b**) CD8 immune response against 122A1 in non-HLA2 patients (TNFα); (**c**) CD8 immune response against 427L in non-HLA2 patients (TNFα); (**d**) CD8 immune response against 331L in non-HLA2 patients (TNFα)

HLA, human leukocyte antigen; TNF, tumor necrosis factor

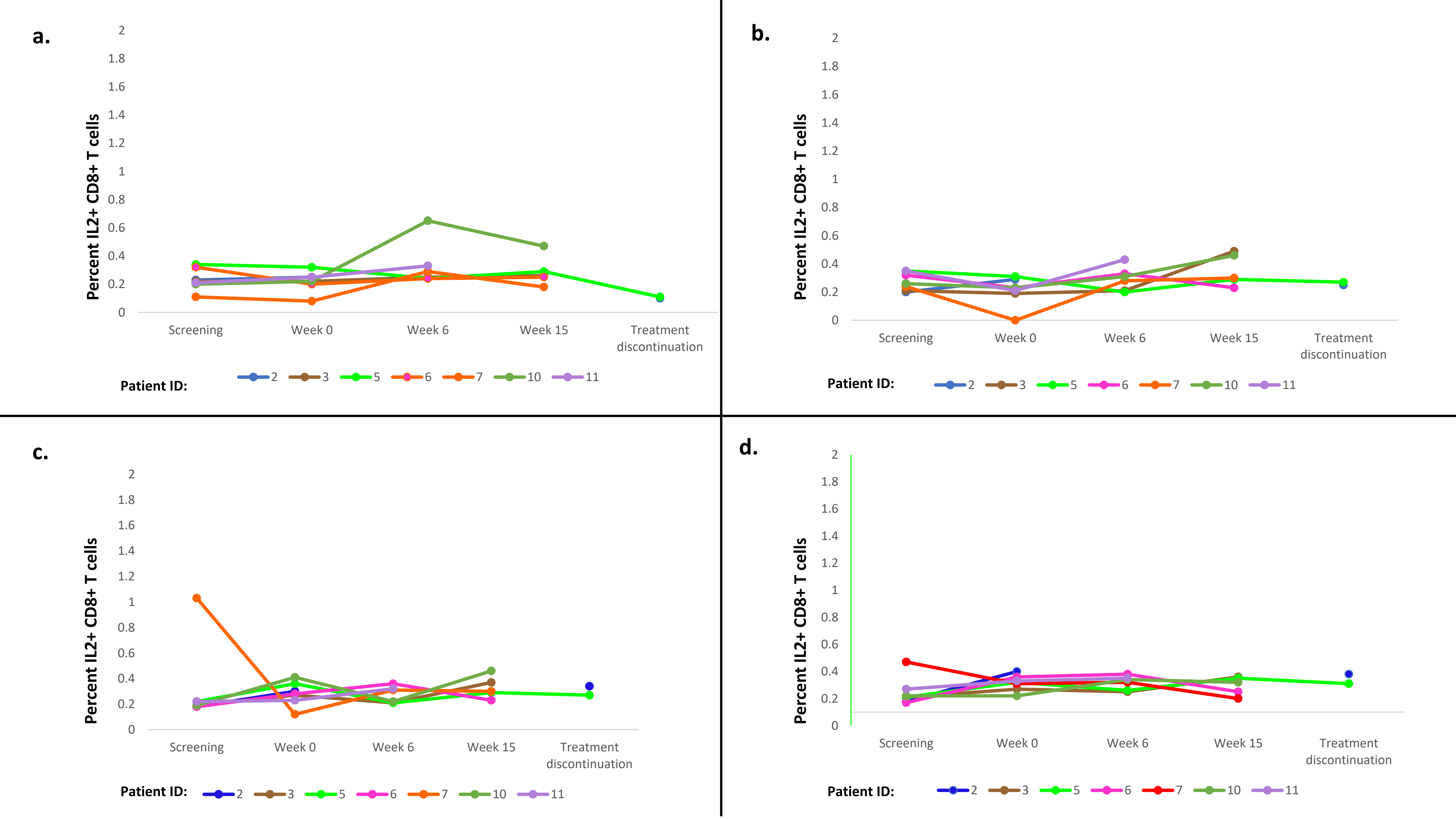
a.
Percent IL2+ CD8+ T cells
2
1.8
1.6
1.4
1.2
1
0.8
0.6
0.4
0.2
0
Screening
Week 0
Week 6
Week 15
Treatment discontinuation
Patient ID:
2
3
5
6
7
10
11
b.
Percent IL2+ CD8+ T cells
2
1.8
1.6
1.4
1.2
1
0.8
0.6
0.4
0.2
0
Screening
Week 0
Week 6
Week 15
Treatment discontinuation
Patient ID:
2
3
5
6
7
10
11
c.
Percent IL2+ CD8+ T cells
2
1.8
1.6
1.4
1.2
1
0.8
0.6
0.4
0.2
0
Screening
Week 0
Week 6
Week 15
Treatment discontinuation
Patient ID:
2
3
5
6
7
10
11
d.
Percent IL2+ CD8+ T cells
2
1.8
1.6
1.4
1.2
1
0.8
0.6
0.4
0.2
0
Screening
Week 0
Week 6
Week 15
Treatment discontinuation
Patient ID:
2
3
5
6
7
10
11

Figure S7: Cytokine responses of CD8 T cells to test peptides. (**a**) CD8 immune response against 122A in non-HLA2 patients (IL-2); (**b**) CD8 immune response against 122A1 in non-HLA2 patients (IL-2); (**c**) CD8 immune response against 427L in non-HLA2 patients (IL-2); (**d**) CD8 immune response against 331L in non-HLA2 patients (IL-2)

HLA, human leukocyte antigen; IL-2, interleukin 2

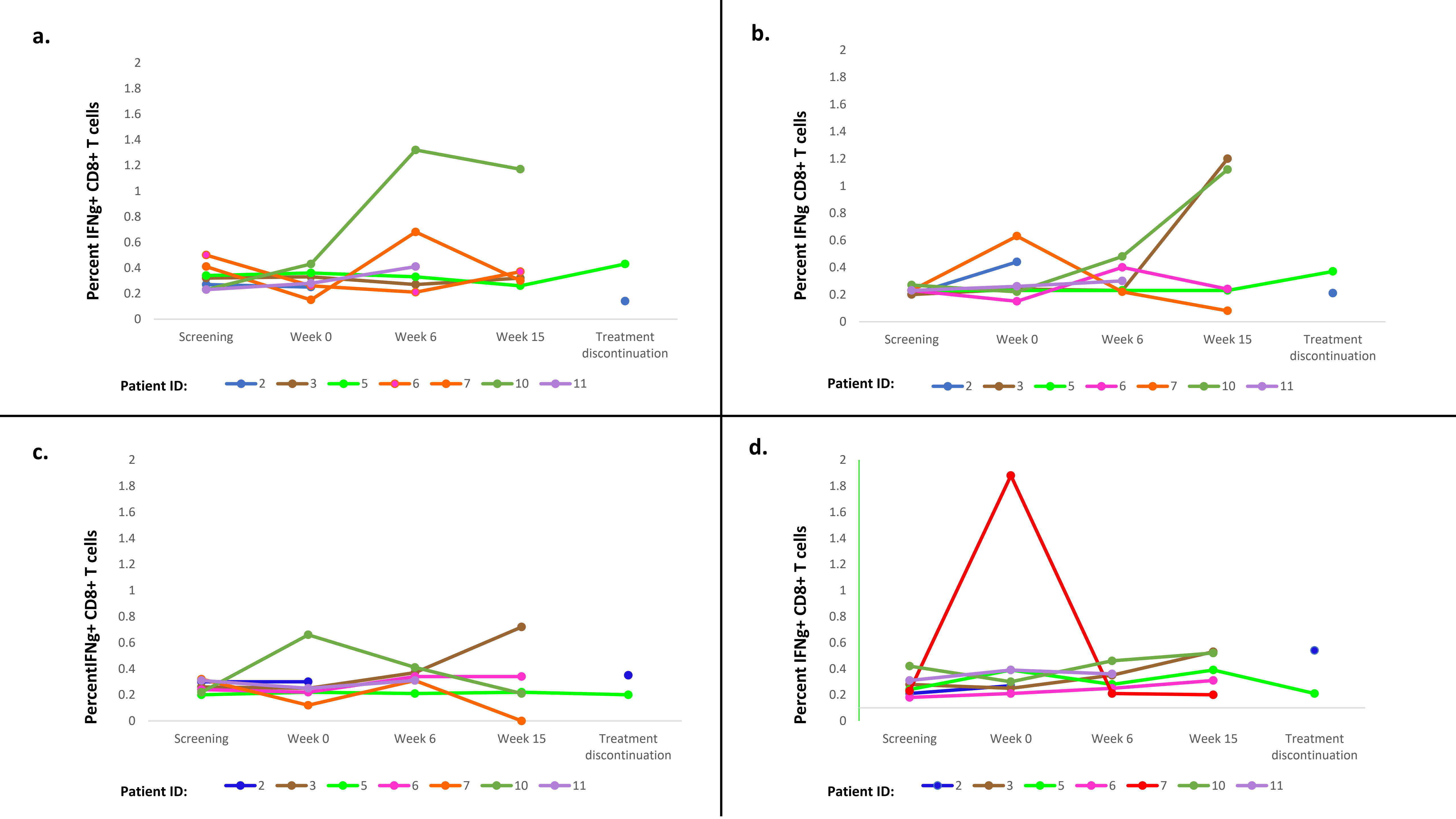
a.
Percent IFNg+ CD8+ T cells
2
1.8
1.6
1.4
1.2
1
0.8
0.6
0.4
0.2
0
Screening
Week 0
Week 6
Week 15
Treatment discontinuation
Patient ID:
2
3
5
6
7
10
11
b.
Percent IFNg CD8+ T cells
2
1.8
1.6
1.4
1.2
1
0.8
0.6
0.4
0.2
0
Screening
Week 0
Week 6
Week 15
Treatment discontinuation
Patient ID:
2
3
5
6
7
10
11
c.
PercentIFNg+ CD8+ T cells
2
1.8
1.6
1.4
1.2
1
0.8
0.6
0.4
0.2
0
Screening
Week 0
Week 6
Week 15
Treatment discontinuation
Patient ID:
2
3
5
6
7
10
11
d.
Percent IFNg+ CD8+ T cells
2
1.8
1.6
1.4
1.2
1
0.8
0.6
0.4
0.2
0
Screening
Week 0
Week 6
Week 15
Treatment discontinuation
Patient ID:
2
3
5
6
7
10
11

Figure S8: Cytokine responses of CD8 T cells to test peptides. (**a**) CD8 immune response against 122A in non-HLA2 patients (IFNɣ); (**b**) CD8 immune response against 122A1 in non-HLA2 patients (IFNɣ); (**c**) CD8 immune response against 427L in non-HLA2 patients (IFNɣ); (**d**) CD8 immune response against 331L in non-HLA2 patients (IFNɣ)

HLA, human leukocyte antigen; IFNɣ, interferon gamma

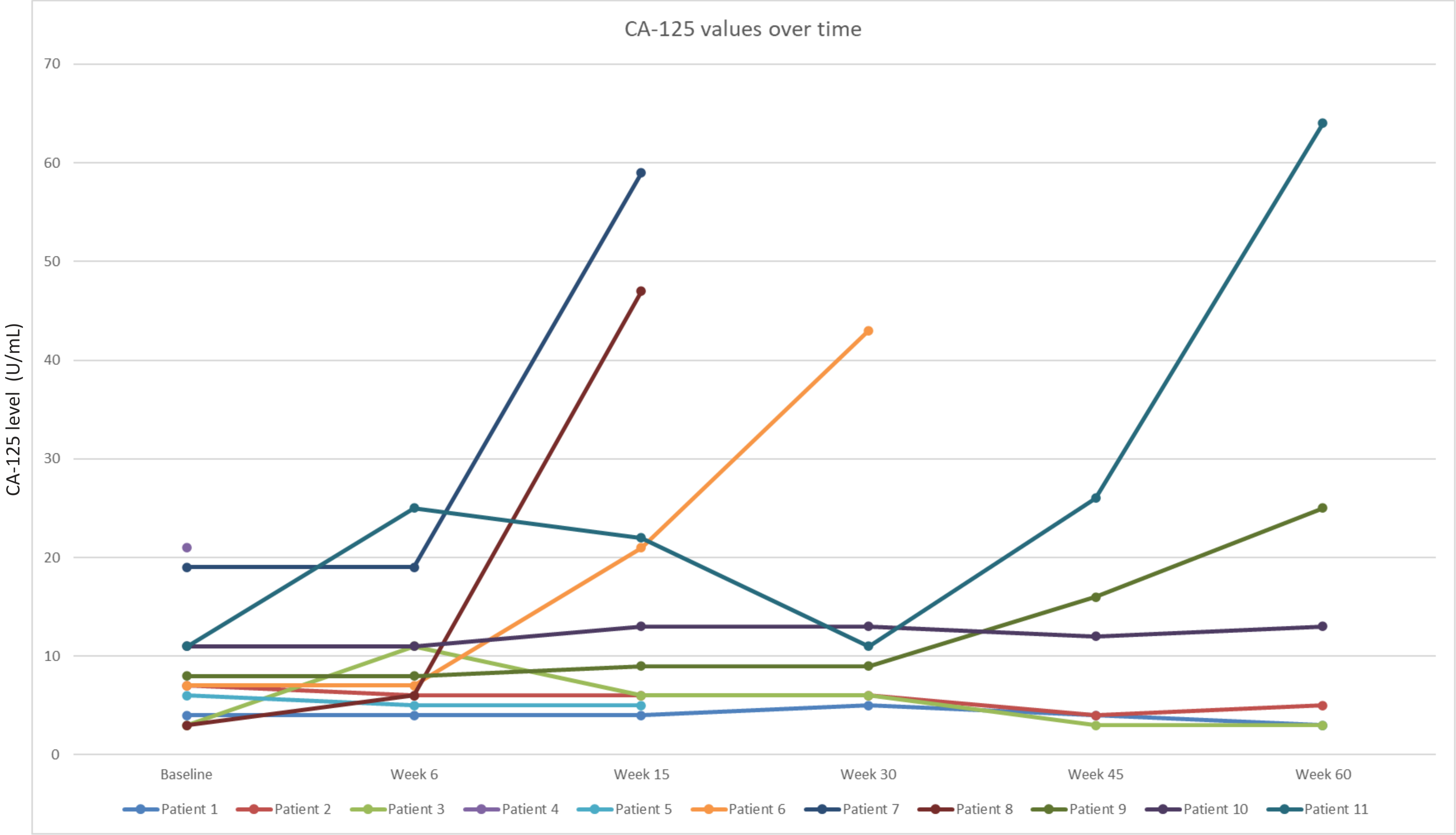

Figure S9: CA-125 responses

Table S1: HLA typing results

Study ID	A	B	C	DRB1	DQB1
1	02:01<br>30:01	41:01<br>45:01	17:MN<br>16:01	04:03<br>07:01	03:05<br>03:03
2	01:01<br>01:01	08:01<br>37:01	07:AJKDW<br>06:DDAR	03:01<br>10:01	02:01<br>05:01
3	24:02<br>31:01	35:08<br>38:01	04:AMEKR<br>12:AJUHH	11:01<br>14:04	03:01<br>05:03
5	23:AFCTD<br>25:01	18:RRG<br>49:01	12:AJUHH<br>07:AJKDW	15:01<br>16:01	06:02<br>05:02
6	11:01<br>33:03	15:01<br>44:03	14:03<br>04:AMEKR	04:06<br>13:02	03:02<br>06:04
7	03:01<br>03:01	08:01<br>35:01	07:01<br>04:01	01:01<br>03:01	05:01<br>02:01
8	02:01<br>30:02	15:10<br>42:01	03:04<br>17:01	13:03<br>14:54	03:01<br>05:03
9	02:01<br>32:01	14:01<br>35:08	08:02<br>04:01	07:01<br>11:04	02:02<br>03:01
10	01:01<br>01:01	35:02<br>52:01	06:02<br>12:02	11:04<br>15:02	03:01<br>06:01
11	24:02<br>24:02	27:05<br>35:02	07:01<br>04:01	11:04<br>14:54	03:01<br>05:03
12	02:01<br>11:01	44:03<br>52:01	16:01<br>12:02	07:01<br>15:AMKZA	02:02<br>06:01
